# FIGO position statement on surrogacy: Ethical considerations

**DOI:** 10.1002/ijgo.70507

**Published:** 2025-09-16

**Authors:** Rafal Zadykowicz, Katie Watson, Aris Antsaklis, the FIGO Committee on Ethical Aspects of Human Reproduction and Women's Health, Edgar Mocanu, Louise Hull, Scott Nelson, Dov Feldberg, Jaideep Malhotra, Goknur Topcu, Akira Iwase, Togas Tulandi, Ajey Bharadwaj, Eytan Barnea, Hrishikesh Pai, Ivon Diaz Yamal, Nikhil Purandare, Carlos Salazar, Lourdes Capito, Ana G. Quijada, Suchitra N. Pandit, Francis G. Muriithi, Fuminori Taniguchi, Alex Vidaeff, Julie Chor, Salome Maswime, Adeline Boatin, Ravi Chandran

**Affiliations:** ^1^ Department of Obstetrics, Perinatology, Gynaecology and Reproduction Medical University of Warsaw Warsaw Poland; ^2^ Departments of Medical Education, Medical Social Sciences, and Obstetrics and Gynecology Northwestern University Feinberg School of Medicine Chicago Illinois USA; ^3^ Department of Obstetrics and Gynecology, School of Medicine, IASO Maternity Hospital University of Athens Athens Greece

**Keywords:** ethical aspects and standards, FIGO, surrogacy

## Abstract

Surrogacy raises complex social and ethical issues related to autonomy, informed consent, potential exploitation, and the welfare of all parties involved. In this article, FIGO advocates for ethically sound surrogacy practices that prioritize voluntary and informed consent, safeguard the well‐being of surrogates, uphold the rights and responsibilities of intended parents, and place the welfare of the child at the center of all decisions. FIGO discourages surrogacy arrangements in which a woman agrees in advance to surrender a child to whom she is genetically related (“traditional surrogacy”) and supports surrogacy arrangements in which a woman agrees in advance to surrender a child to whom she is not genetically related (“gestational surrogacy”) provided they align with core ethical principles. FIGO also emphasizes the importance of adhering to international ethical standards, particularly in the context of cross‐border surrogacy arrangements.

## 1 INTRODUCTION

Surrogacy, the practice in which a woman (the surrogate) agrees to bear a child for another person or couple (the intended parents), has long been the subject of ethical debate and legal complexity worldwide.

Although surrogacy arrangements can be traced back to ancient civilizations, the modern practice emerged in the 1970s and 1980s, with the first reported case of gestational surrogacy occurring in the USA in 1985.^1^, ^2^

Legislative responses to surrogacy began in earnest in the 1980s. The UK introduced the Surrogacy Arrangements Act of 1985 after the public outcry surrounding the “Baby Cotton” case.^3^ The Act sought to regulate surrogacy by prohibiting commercial arrangements while allowing altruistic ones. During this period, courts also addressed surrogacy disputes in jurisdictions lacking clear legislation. In the USA, for instance, the New Jersey Supreme Court prohibited traditional surrogacy in the Baby M case (1988), and the California Supreme Court allowed gestational surrogacy in Johnson v. Calvert (1993).

Globally, legal approaches to surrogacy vary widely, reflecting diverse cultural, ethical, and social norms. Countries like India, Canada, Ukraine, and Russia, as well as most states within the USA, have established legal frameworks that explicitly permit or regulate surrogacy, including commercial arrangements. In contrast, countries such as France, Germany, Spain, and Italy strictly prohibit all forms of surrogacy on ethical and moral grounds.^4^, ^5^ Others, like Australia and the UK, allow only altruistic surrogacy and prohibit commercial surrogacy to avoid exploitation and the commodification of reproduction.^6^, ^7^

Religion also plays a significant role in shaping surrogacy laws. Various religious traditions have differing views on the ethics of assisted reproductive technologies. In Islamic jurisprudence, interpretations vary—some allow surrogacy under specific conditions, while others prohibit it entirely, contributing to restrictive policies in many Muslim‐majority countries. Catholicism generally opposes surrogacy, viewing it as a separation of procreation from the conjugal act, influencing restrictive laws in Catholic‐majority countries. In contrast, Judaism often adopts a more permissive stance, particularly under specific ethical and halachic conditions, contributing to more accommodating frameworks in countries like Israel.^8^, ^9^, ^10^

This international diversity in legal and ethical approaches underscores the ongoing global debate over surrogacy. The International Federation of Gynecology and Obstetrics (FIGO) focuses on the ethical aspects of surrogacy, advocating for the rights, well‐being, and autonomy of all involved parties, while refraining from taking positions on the specific legal frameworks that differ across jurisdictions. FIGO emphasizes that anyone capable of pregnancy must be treated with respect, dignity, and autonomy.^1^, ^2^, ^11^, ^12^

## 2 TYPES OF SURROGACY

Surrogacy generally falls into two categories: traditional and gestational.

Traditional surrogacy involves a surrogate who is genetically related to the child. Conception occurs through artificial insemination using the intended father's sperm or donor sperm. As a result, the surrogate is both the genetic and gestational mother. This arrangement raises distinct ethical, legal, and emotional concerns, particularly surrounding the genetic and emotional bonds between the surrogate and the child.^13^, ^14^

Gestational surrogacy, by contrast, involves the implantation of an embryo created via in vitro fertilization (IVF), using the gametes (egg and/or sperm) of the intended parents or donors. In this form, the surrogate has no genetic connection to the child and serves solely as a gestational carrier. It has become increasingly common and is often viewed as less ethically complex due to the absence of genetic ties between the surrogate and the child.^15^, ^16^

Although the ethical principles below apply to both types of surrogacy, gestational surrogacy raises fewer ethical concerns compared to traditional surrogacy, where genetic ties complicate issues of consent, parenthood, and potential exploitation. Traditional surrogacy should be clearly distinguished from arrangements that resemble adoption and those that risk unethical commercialization or “baby selling.”

Given the complexity of these distinctions, FIGO discourages traditional surrogacy arrangements, particularly those in which a woman agrees in advance to surrender a child to whom she is genetically related. FIGO instead supports gestational surrogacy arrangements, provided they align with core ethical principles, including respect for autonomy, informed consent, non‐exploitation, and prioritization of the child's welfare.^1^, ^2^, ^11^, ^12^, ^17^

## 3 ETHICAL PRINCIPLES

### 3.1 Autonomy: Informed consent

The principle of autonomy is fundamental to ethical medical practice. Both the surrogate and the intended parents must have the autonomy to make informed decisions free from coercion. Informed consent means that the surrogate fully understands the medical, psychological, and social implications of the gestational surrogacy arrangement. This includes potential health risks, emotional impacts, financial considerations, and the legal ramifications of relinquishing parental rights.^4^, ^5^, ^6^, ^18^, ^19^, ^20^

To ensure truly informed and autonomous decision‐making, both the surrogate and the intended parents must receive independent and comprehensive counseling before entering into a surrogacy agreement. This counseling should cover the risks and benefits of the surrogacy process, the medical procedures involved, pregnancy‐related risks (including prenatal diagnosis), and the potential impact of eventual disclosure to the child. The counseling must be factual, non‐coercive, respectful of the individuals' personal views and decisions, and be provided by individuals with appropriate qualifications.

Surrogacy agreements should include contracts that outline the responsibilities of all parties involved. These agreements often require the surrogate to follow health guidelines that minimize risks to the child, such as adhering to nutritional recommendations and participating in prenatal screenings. At the same time, the intended parents are obligated to accept full parental responsibility for the child under any circumstances, including in cases of congenital or genetic abnormalities, or pregnancy and labor‐related neonatal complications. These provisions help ensure that autonomy and informed consent are upheld, while also protecting the rights and welfare of all involved parties.^1^, ^7^, ^11^, ^21^

### 3.2 Non‐maleficence and justice: Protecting against exploitation and coercion

One of the most significant ethical concerns is the potential exploitation of surrogates, particularly in economically disadvantaged situations. Economic or other types of coercion invalidates consent. Surrogacy agreements must ensure that surrogates are not coerced by financial incentives or social pressures. Ethical surrogacy practices require fair compensation that reflects the surrogate's service without exploiting their socioeconomic status. In altruistic surrogacy arrangements, where compensation is limited strictly to reimbursement of expenses, particular attention should be given to ensure that decisions are genuinely voluntary, free from coercive emotional or familial pressures, and fully informed. Manipulation and misunderstanding also invalidate consent.^22^ Ethical surrogacy practice requires that surrogates have independent legal advice to ensure that they have an accurate understanding of their legal rights and responsibilities, and the true meaning of any contracts they may be asked to sign.^20^, ^23^

### 3.3 Beneficence: Health and well‐being of the surrogate

The surrogate's health and well‐being must be prioritized. This includes comprehensive healthcare access, regular medical check‐ups, and psychological support throughout the pregnancy and postpartum period. The physical and mental health risks associated with pregnancy and childbirth should be clearly communicated, and support systems should be in place to manage any complications. The surrogate must remain the ultimate decision‐maker on the mode of delivery and any maternal interventions offering informed consent or informed refusal to any medical procedure.^24^ Furthermore, all efforts must be undertaken to reduce the chance of multiple pregnancy with the ensuing risk to the surrogate mother and future children.^12^, ^25^

The surrogate's psychological and emotional health needs beyond her pregnancy must also be prioritized. For example, it is unjust to require a surrogate to stop seeing and caring for her existing children when doing so does not pose a significant threat to her pregnancy. This approach aligns with established legal and ethical frameworks, such as those outlined in the UK's Surrogacy Arrangements Act 1985 and subsequent Human Fertilization and Embryology Act 2008, which emphasize the surrogate's autonomy and dignity. UK law explicitly recognizes the surrogate as the legal mother at birth and thus implicitly safeguards her right to manage her familial relationships and caregiving responsibilities throughout pregnancy unless there is clear evidence of significant risk.^26^, ^27^ In the USA, the law of the state of Illinois also emphasizes the surrogate's autonomy and dignity—for example, it requires that she have delivered at least one child to be eligible for surrogacy to ensure she knows what pregnancy and potential separation would be like—but if all statutory requirements are met, then the names of the intended parents go on the birth certificate.^28^

### 3.4 Beneficence: Rights and welfare of the child

The welfare of the child born through surrogacy must be a paramount consideration. Ethical considerations include ensuring the child's right to know their genetic origin and protection against any form of discrimination or stigmatization.^29^, ^30^, ^31^ The best welfare of the child should be a guiding principle in all surrogacy arrangements. The rights of the child born through surrogacy to know the identity of their birth parents should be explicitly recognized and safeguarded, given the profound implications for the child's identity, sense of self‐knowledge, well‐being, and belonging.

### 3.5 Non‐maleficence: Psychological impact

Surrogacy can have significant psychological impacts on the surrogate, the intended parents, and the child.^32^, ^33^ Psychological screening and ongoing counseling should be mandatory to support all parties involved. This includes addressing the potential emotional challenges faced by surrogates in relinquishing the child, as well as those by intended parents in terms of bonding and attachment.

### 3.6 Ethical considerations for cross‐border surrogacy

Cross‐border surrogacy, where intended parents seek surrogates in other countries, often due to lower costs or legal restrictions in their home countries, raises additional ethical concerns. These include disparities in healthcare standards, insufficient legal protections for surrogates, and the potential for exploitation. Ethical surrogacy practices require international cooperation and adherence to universally accepted ethical standards.^34^, ^35^, ^36^

## 4 CONCLUSION

Surrogacy presents complex ethical challenges that require a balanced approach—one that respects the rights and well‐being of the surrogate, the intended parents, and the child. FIGO discourages traditional surrogacy, and advocates for gestational surrogacy practices rooted in informed consent, autonomy, non‐exploitation, and the best interests of all parties involved. Ongoing research, thoughtful policy development, and international collaboration are essential to address the evolving ethical landscape of surrogacy.

FIGO recommends that all countries adopt legislation designed to protect the interests of everyone involved. Such legislation should provide clear guidance for resolving potential disputes, with the welfare of the future child placed at the center of all decision‐making.

This FIGO position statement underscores the importance of ethically sound gestational surrogacy practices that uphold the dignity, rights, and health of all participants. It aims to offer a framework for medical professionals and policymakers navigating the complex ethical dimensions of surrogacy.

### 4.1 FIGO recommendations on ethical surrogacy practices

1. Gestational surrogacy arrangements should be supported when aligned with ethical principles, including respect for autonomy, informed consent, non‐exploitation, and prioritization of the future child's welfare.
2. All surrogacy agreements must explicitly prevent exploitation and coercion by ensuring fair compensation and humane contract terms without capitalizing on socioeconomic vulnerabilities. Surrogates must have access to independent legal advice to fully understand their rights and responsibilities.
3. Comprehensive health care, including physical, psychological, and emotional support, must be provided to the surrogate throughout pregnancy and postpartum. The surrogate should retain autonomy regarding decisions on medical procedures and mode of delivery. Efforts should be made to minimize risks associated with multiple pregnancies.
4. The welfare and best interests of the future child must remain central to all surrogacy arrangements. Children have the right to know their genetic and birth origins, and protection from discrimination and stigmatization must be explicitly ensured.
5. Cross‐border surrogacy arrangements require international regulatory frameworks to ensure ethical standards, equitable health care, robust legal protections for surrogates, and protection against exploitation and coercion.

## AUTHOR CONTRIBUTIONS

RZ wrote the initial draft of the manuscript. All authors made substantial contributions to the conception or design of the work, reviewed the paper critically for content, and approved and agreed to be accountable for the final published version.

## CONFLICT OF INTEREST STATEMENT

The authors have no conflicts of interest.

## MEMBERS OF THE FIGO COMMITTEE ON ETHICAL ASPECTS OF HUMAN REPRODUCTION AND WOMEN'S HEALTH, 2023–2025

Aris Antsaklis (Chair), Carlos Salazar (Vice Chair), Lourdes Capito, Rafal Zadykowicz, Ana G. Quijada, Suchitra N. Pandit, Francis G. Muriithi, Fuminori Taniguchi, Alex Vidaeff, Julie Chor, Salome Maswime, Adeline Boatin, Katie Watson, Ravi Chandran.

## Data Availability

Data sharing is not applicable to this article as no new data were created or analyzed in this study.
